# Acquired non-compaction in integrin-myopathy

**DOI:** 10.1186/1750-1172-8-183

**Published:** 2013-11-20

**Authors:** Josef Finsterer, Sinda Zarrouk-Mahjoub

**Affiliations:** 1 Krankenanstalt Rudolfstiftung, Postfach 20, 1180 Vienna, Austria; 2 Laboratory of Biochemistry, UR “Human Nutrition and Metabolic Disorders” Faculty of Medicine Monastir, Monastir, Tunisia

## Letter to the Editor

With interest we read the article by Esposito et al. about a female child with myopathy and left ventricular hypertrabeculation / noncompaction (LVHT) carrying a mutation in two different genes, the integrin-α7 gene and the myosin heavy chain 7B gene (MYH7B) [1]. We have the following comments and concerns.

The authors mention in the discussion that MYL2 and MYL3 mutations were found in patients with LVHT [1]. Among the 5 papers cited to corroborate this statement Budde et al. [2] did not look for MYL2 or MYL3 mutations and Klaassen et al. [3] and Probst et al. [4] definitively state that no mutations in MYL2 or MYL3 were found. Hoedemaekers et al. 2010 [5] tested for MYL2 and MYL3 but do not mention a mutation in these genes in their results either. Walsh et al. 2010 [6] is not an original paper but a review about MYH7 mutations.

LVHT has not only been found in association with mutations in the TAZ, DTNA, ZASP, lamin A/C, MYH7, ACTC1, TNNT2, TNNI3, MYBPC3, and TPM1 genes but also in association with mutations in the dystrophin, DMPK, ZNF9, LAMP2, GAA, mtDNA, AMPD1, GBE1, RYR1, COL7A1, PMP22, MMACHC, beta-globin, and DNAJC19 genes [Finsterer et al., submitted].

The index patient underwent echocardiography at age 1 month but LVHT was diagnosed not before the next echocardiographic examination at age 17 months. Which is the reason why LVHT was not detected at the initial examination? Was LVHT missed because of poor image quality, absent awareness of the pathology, ignoring the abnormality, left ventricular hypertrophy at the initial investigation, severe dilation of the left ventricle, or did LVHT truly develop during the period between the two examinations (acquired LVHT)? Did the authors review the initial echocardiographic examination? Did the presence of a patent arterial duct and patent foramen ovale prevent LVHT from being diagnosed at the initial examination?

Myopathy is often subclinical at an early stage and may be detected or suspected only upon creatine-kinase (CK) screening, needle electromyography or muscle biopsy at this stage [7]. Were the three other females with LVHT or other family members seen by a myologist? Did they report any symptoms indicative of a muscle disease? Were any signs found which suggested the presence of a neuromuscular disorder? Which were the CK values in these three individuals? Since all family members underwent needle electromyography and muscle biopsy it would be worthwhile to know if these investigations were abnormal in any of them as well.

The patient obviously died from sudden cardiac death, which is a frequent complication in patients with LVHT. Were there any other family members of the 5 generation family in whom sudden cardiac death has been reported? Since ECGs had been recorded in all family members it would be interesting to know if QTc was prolonged in any member other than the index patient? Was implantation of an implantable cardioverter defibrillator ever considered in case V/4?

Since LVHT may be also complicated by cardioembolic events, death could be attributable also to fatal cerebral or cardiac embolism [8]. Did the patient undergo autopsy including the cerebrum to exclude such a cause?

The authors mention that the index patient underwent cardiac MRI at age 10y. Was any late enhancement observed and if there was late enhancement how was it distributed?

Overall, the interesting paper by Esposito et al. evokes a number of questions, which require further discussion. Currently, there is no proof that LVHT is causally linked to any of the mutations so far described in association with LVHT.
